# 5-aminolevulinic acid induced photodynamic reactions in diagnosis and therapy for female lower genital tract diseases

**DOI:** 10.3389/fmed.2024.1370396

**Published:** 2024-07-15

**Authors:** Yuqing Chen, Peng Guo, Lihong Chen, Dalin He

**Affiliations:** ^1^Department of Urology, The First Affiliated Hospital of Xi’an Jiaotong University, Xi'an, Shaanxi, China; ^2^Department of Obstetrics and Gynecology, Shaanxi Provincial People’s Hospital, Xi'an, Shaanxi, China

**Keywords:** 5-aminolaevulinic acid, photodynamic therapy, photodynamic diagnosis, HPV infection, female lower genital tract diseases

## Abstract

Since the patients suffering from female lower genital tract diseases are getting younger and younger and the human papilloma virus (HPV) infection is becoming more widespread, the novel non-invasive precise modalities of diagnosis and therapy are required to remain structures of the organ and tissue, and fertility as well, by which the less damage to normal tissue and fewer adverse effects are able to be achieved. In all nucleated mammalian cells, 5-Aminolevulinic acid (5-ALA) is an amino acid that occurs spontaneously, which further synthesizes in the heme biosynthetic pathway into protoporphyrin IX (PpIX) as a porphyrin precursor and photosensitizing agent. Exogenous 5-ALA avoids the rate-limiting step in the process, causing PpIX buildup in tumor tissues. This tumor-selective PpIX distribution after 5-ALA application has been used successfully for tumor photodynamic diagnosis (PDD) and photodynamic therapy (PDT). Several ALA-based drugs have been used for ALA-PDD and ALA-PDT in treating many (pre)cancerous diseases, including the female lower genital tract diseases, yet the ALA-induced fluorescent theranostics is needed to be explored further. In this paper, we are going to review the studies of the mechanisms and applications mainly on ALA-mediated photodynamic reactions and its effectiveness in treating female lower genital tract diseases.

## 1 Introduction

Light, lasers, renewable energy, and other technologies have apparently captured the attention of the globe. Thus, it is important to remember that the light not only exists at the beginning of everything, but it also has the ability to both nurture and kill. One of the effects induced by light that occupies an important position is the photodynamic reactions.

Porphyrins, especially protoporphyrin IX (PpIX), are abundant in nature and, depending on the metal type, perform a variety of activities (1). Recent research has revealed that the buildup of PpIX in abnormal cells is related to the uptake and efflux transporters, albeit the mechanism is unknown (2). The 5-aminolevulinic acid (5-ALA), with no fluorescence, is an endogenous agent that involves in the biochemical chain metabolizing PpIX which is non-toxic and naturally occurs in the body, and emits red fluorescence (650 nm) with the irradiation of blue excitation light (400 nm), and further induces photodynamic reactions basically including photodynamic diagnosis and photodynamic therapy.

Photodynamic diagnosis (PDD) depends on the topical or systemic application of 5-ALA stimulating the accumulation of endogenous photosensitizer PpIX in diseased cells (3), and the generated fluorescence will assist in the localization of PpIX-rich areas and identification of the feasible cancerous tissues. Additionally, the buildup PpIX is capable of mediating photodynamic therapy, in which the singlet oxygen and reactive oxygen species are generated with the oxygen and the irradiation light of a certain wavelength. And these cytotoxic substances are able to damage diseased cells directly and indirectly. Based on the local cytotoxic reactions, PDT is used as a promising treatment for lots of diseases reducing severe side effects and complications in excisional and destructive modalities (4). The therapeutic effect of PDT is well-restricted in the area generating fluorescence, along with the short-lived cytotoxic species, ensures the local damage happening in the malignant lesions without sparing to the normal adjacent tissues (5). During conventional treatments, the produced fluorescence can also be used for evaluating the exact boundaries of the lesion as a guidance for the resection. Despite the well-recognized use in treatment of tumors, the photodynamic effect was initially observed against microorganisms around the turn of the twentieth century. In view of antibiotic resistance and the prevalence of new diseases, photodynamic inactivation (PDI) of microorganisms (bacteria, fungi, and viruses) deserves great consideration (6). Regarding to the close relationship between the gynecological malignant lesions and the infection of human papilloma virus (HPV), ALA-PDI may also make a critical contribution to the killing of viruses directly.

Used ALA-PDT in treating basal cell carcinoma, Kennedy et al. reported the first application of PDT in 1990 (7). Since then, ALA-PDD and ALA-PDT have been applied for numerous diseases, such as actinic keratosis, Bowen’s disease (8), bladder cancer and brain tumor (9–11), etc. Because of the effective role played by ALA-PDT and ALA-PDT on HPV infection, they have made a great breakthrough in treating some reproductive tract diseases, like cervical intraepithelial neoplasia (CIN), vulva intraepithelial neoplasia (VIN), vaginal intraepithelial neoplasia (VaIN) and condyloma acuminatum (CA) (12).

This review focuses on the mechanisms and applications of ALA-induced photodynamic reactions (primarily ALA-PDD and ALA-PDT) in female lower genital tract diseases, involving mostly researches of the last decade and brings forward the advantages compared with conventional therapy and the existing deficiencies.

## 2 The precursor of PpIX—5-aminolevulinic acid

### 2.1 ALA and its derivative drugs in use

It has been widely agreed that a photosensitizer drug is supposed to have the following qualities: selective tumor absorption, low biotoxicity without irradiation, and long absorption wavelength which ensures itself to penetrate deeper tissue (13). Several photosensitizer drug investigated involve hypericin (14), porfimer (15), and 5-aminolevulinic acid (5-ALA) (16). Among them, the prodrug ALA and its ester derivatives with reinforced lipophilicity—methyl (Me-ALA) and hexyl (He-ALA) esters are the most popular porphyrin-related photosensitizers for PDT and photodiagnosis (Table 1) (17). As drugs for PDT, they are widely used in treatments for superficial skin lesions such as actinic keratosis, basal cell carcinoma and Bowen’s disease. For photodynamic diagnosis (PDD), they are capable of decide malignant lesions through fluorescence emission, including bladder tumors and gliomas which benefits the tumor diagnosis and resection with high precision. ALA is produced in the mitochondria of practically all cells and further creates heme in heme biosynthetic pathway, through which a kind of fluorescent substance—protoporphyrin IX (PpIX) is generated meanwhile exhibiting red fluorescence and inducing photosensitizing activity with light excitation. The application of ALA as the prodrug for PpIX-induced PDT and PDD is based on the fact that exogenous ALA absorbed by tumor cells is able to mediate the biosynthesis of PpIX.

**Table 1:** The clinical application of ALA and its derivatives.

Prodrug	Ex wave-length	Indications
ALA	635 nm	Actinic keratoses and squamous cells carcinoma in dermatology
		Mycosis fungoides
		Bladder and upper urinary tract carcinoma, prostate cancer
		Squamous cells carcinoma and high-grade dysplasia in head and neck
		Leukoplakia, erythroplakia, erythro-leukoplakia and verrucous hyperplasia
		Purging of leukemic cells in autologous bone marrow transplantation in leukemia
		Barrett’s esophagus, esophagus and gastric cancer
		Oral malignancies photodetection
		FGR in gliomas, meningiomas, brain metastases and pediatric brain tumors
		Fluorescence hysteroscopy for endometrial cancer
		Fluorescence-guided second look laparoscopy for ovarian cancer metastasis detection
		Gastric cancer and metastatic lymph nodes detection
		Barrett’s esophagus dysplasia monitoring
		Peritoneal carcinomatosis and metastases detection
		Leukemic cells detection
Hexyl-ALA	635 nm	Actinic keratoses, basal cell carcinoma, and squamous cells carcinoma
		Mycosis fungoides
		Bowen’s disease
		Fluorescence cystoscopy for diagnosis of non-muscle invasive papillary bladder cancer
Methyl-ALA	579–670 nm	Actinic keratoses, basal cell carcinoma in dermatology
		Intraepithelial neoplasias in gynecology, extramammary Paget disease
		Chest wall breast cancer
		Squamous cells carcinoma in head and neck
		Laryngeal carcinoma, lung and pleural cancer detection
		Bladder and upper urinary tract cancer diagnosis and FGR
		Prostate and renal cancer FGR
		Fluorescence-guided laser therapy for penile carcinoma and precancerous lesions
		Sentinel lymph node detection of breast cancer

### 2.2 ALA-induced PpIX biosynthesis

**Figure 1:**
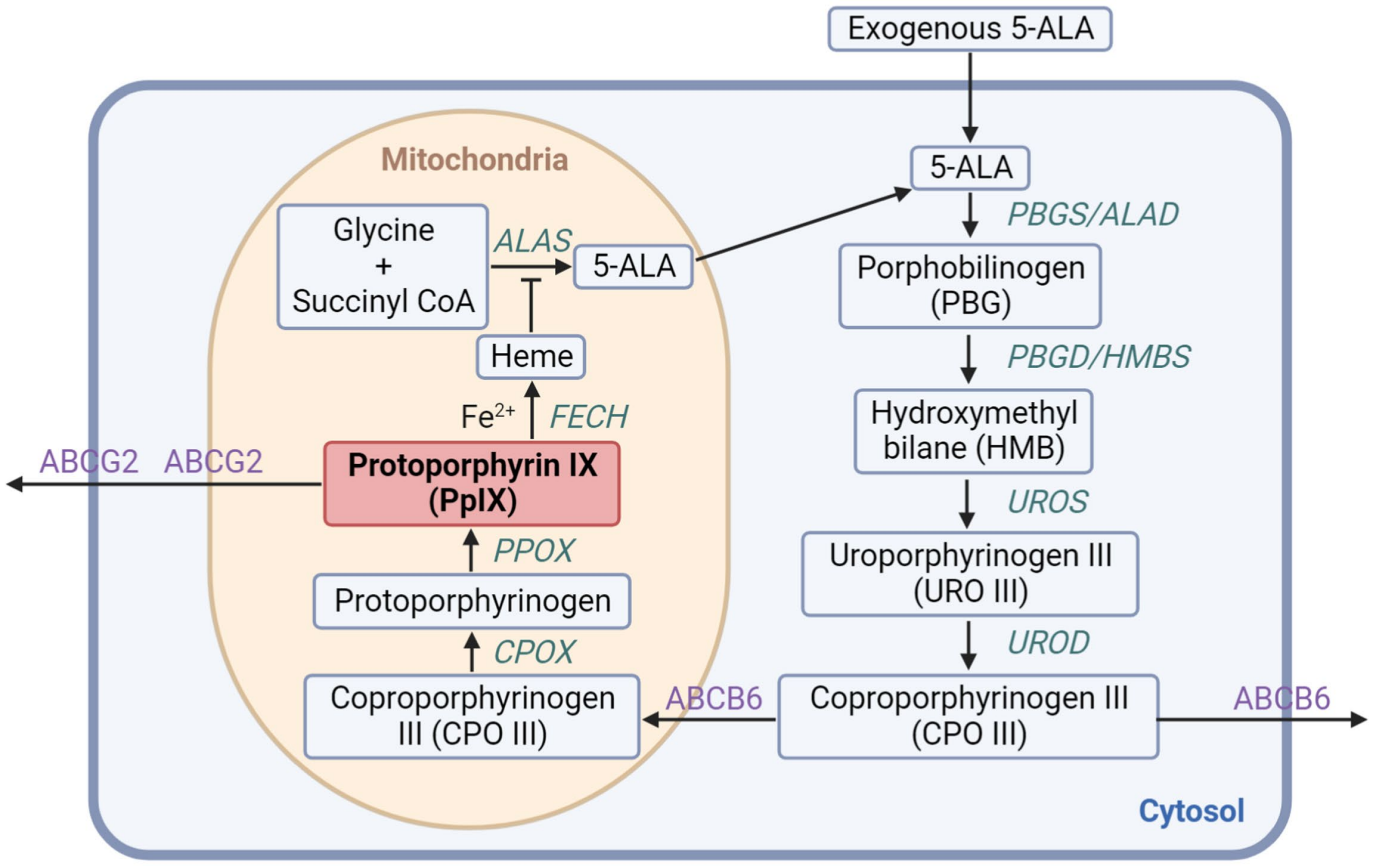
The heme biosynthesis pathway involving 5-ALA and PpIX. The exogenous 5-ALA participates in the biosynthesis of heme with enzymes localized in the cytoplasm and the mitochondrion generating PpIX, which can further be transformed into heme or discharged mainly through ABCG2 transporter.

As shown in Figure 1, eight enzymes are needed for the meticulously regulated process of heme production in mammalian cells, with four of them located in the cytoplasm and the others in the mitochondrion (18). Firstly, through ALA synthase (ALAS), glycine and succinyl-CoA synthesize ALA in the mitochondria, which is the first intermediate in the pathway without fluorescence or photosensitizing activity. As the rate-limiting step in the heme biosynthesis pathway, topically provided exogenous ALA will greatly accelerate the process. Secondly, ALA dehydratase (ALAD) catalyzes two ALA molecules into porphobilinogen synthase (PBGS). Hydroxymethylbilane (HMB) is formed via the deamination of PBG by porphobilinogen deaminase (PBGD), also known as hydroxymethylbilane synthase (HMBS). And then the cyclic tetrapyrrole molecule uroporphyrinogen III (URO III) is going to be produced by uroporphyrinogen III synthase (UROS). Uroporphyrinogen decarboxylase (UROD) subsequently induces the decarboxylation of URO III forming coproporphyrinogen III (CPO III), which is also the last step in the cytosol. Next, in the mitochondrial intermembrane space, CPO III is transformed into protoporphyrinogen IX, which then crosses the mitochondrial inner membrane and generates PpIX, a porphyrin metabolite with fluorescence and photosensitizing activity, via aromatization by protoporphyrinogen oxidase (PPOX). Finally, in the matrix, ferrochelatase (FECH) inserts ferrous iron (Fe^2+^) into PpIX forming heme. Because of the paramagnetic iron added, heme has no fluorescence or photosensitizing activity (19).

### 2.3 PpIX accumulation from ALA in tumor cells

As is known to us all, it is the selective PpIX accumulation in tumor cells that sets the foundation for ALA-mediated PDD and PDT. However, the theory establishing the issue has still remained unclear so far. Though some preceding researches have mentioned that the reduction of FECH activity in tumor cells and the increase of transferrin receptor activity in heme biosynthesis account for the phenomenon (20), recent studies have been unable to figure out the connection between PpIX levels in tumor cells and the expression of FECH or any other specific enzymes in heme biosynthesis pathway and porphyrin transporters (21–23). More interestingly, according to the databases (24), even for some tumors commonly being diagnosed and treated through ALA-mediated PDD and PDT, there is no significantly difference on expression levels of enzymes between tumor and normal tissue, for example, the bladder urothelial carcinoma, which expresses strong fluorescence after rendering exogenously ALA. Probably, a lot of metabolic pathways participate in the selective ALA accumulation in tumor cells, for instance, the glucose and iron metabolism, which are typically changed in tumor cells and closely related to heme biosynthesis.

## 3 Mechanism

### 3.1 ALA-mediated photodynamic diagnosis

**Figure 2:**
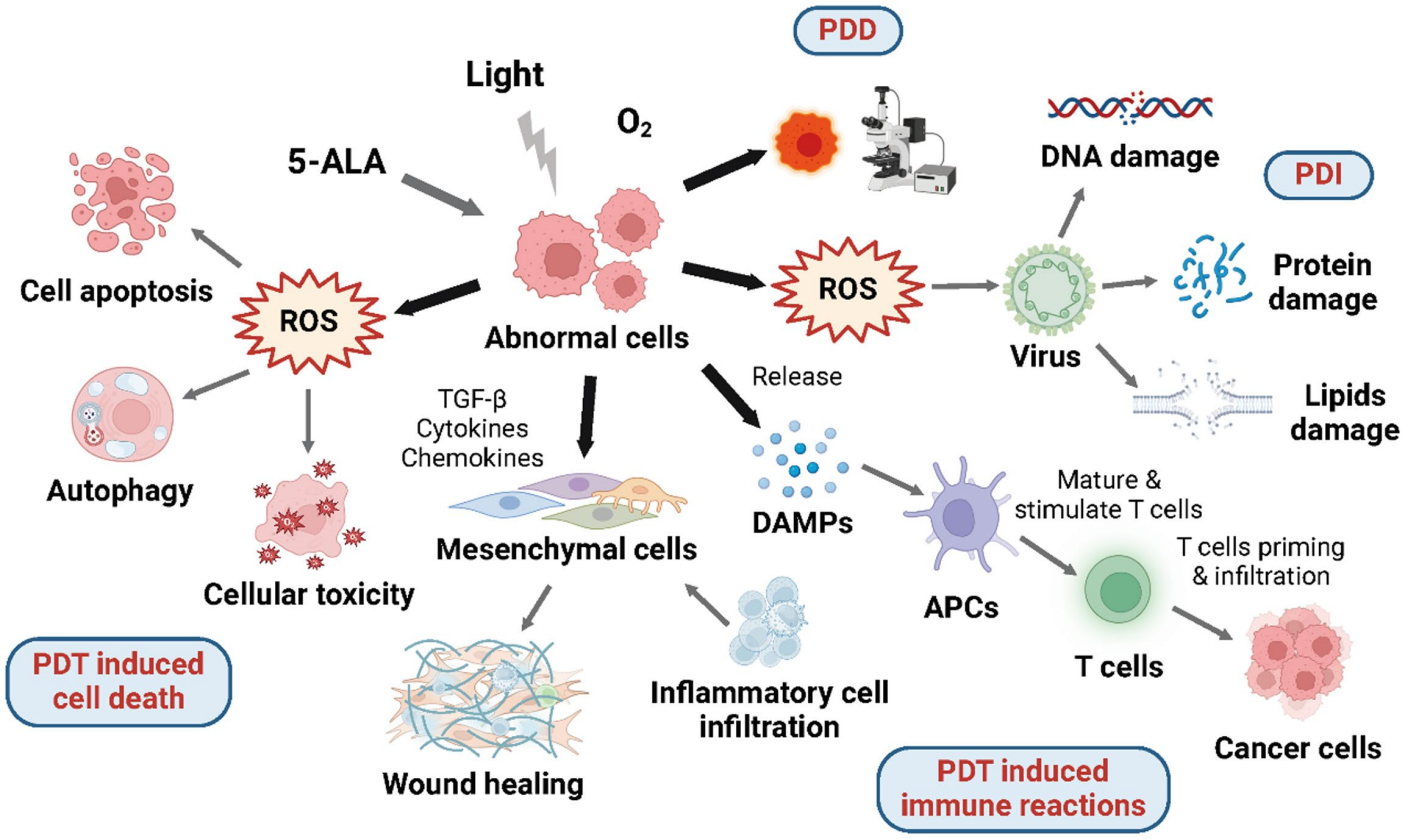
Mechanisms of 5-ALA mediated photodynamic reactions. Under aerobic conditions, 5-ALA and given wavelength of laser will induce photodynamic reactions including PDD, PDT induced cell death, PDT induced immune reactions, and PDI.

As shown in Figure 2, after irradiation, the fluorescent and photosensitizing property of PpIX converted from ALA applied make it the diagnostic probe for malignant lesions, which is called photodynamic detection mediated by ALA (ALA-PDD). PpIX has many absorption peaks in the visible Q-bands and a maximal absorption peak in the soret band at around 405 nm, which allows it to be excited by longer light wavelengths to penetrate deeper into the tissue (25). After being excited by violet/blue light (375–475 nm), PpIX exhibits a wide spectrum of red fluorescence that starts at around 635 nm and goes beyond 700 nm. ALA has been utilized as an intraoperative probe to assist tumor excision because ALA-induced PpIX fluorescence in lesions can afford surgeons a real-time guidance for tumor detection and margin estimation (26).

### 3.2 ALA-mediated photodynamic therapy

Photodynamic therapy (PDT) is a type of light-based therapy using molecular oxygen, a photosensitizer, and a light source with a certain wavelength which may lead to cell death and immune reactions (Figure 2). Although each of these elements by itself is harmless, they become cytotoxic once organized because of the production of reactive oxygen species (ROS) through type I and type II photochemical processes. Photosensitizer molecules lying on the ground singlet state are triggered to the excited singlet state after absorbing light energy, which has a rather ephemeral lifespan and will either experience intersystem transforming to the excited triplet state, or decline back to the ground state via energy dissipation by the emission of fluorescence and conversion to heat. In the Type I reaction, photosensitizer molecules at the triplet state can transfer electrons to substrate molecules straightway creating free radicals; in the Type II reaction, which is more common and frequently occurs in tissues consuming oxygen, the energy is transferred to molecular oxygen producing single oxygen (^1^O_2_). Singlet oxygen has a high degree of reactivity, making it capable of oxidizing a wide range of biological components, such as proteins, lipids, and nucleic acids, which do harm to the tissue. The location of oxidative impairment is near to the part where the photosensitizer applied or produced because of the short lifespan and diffusion distance of ^1^O_2_ (27). Several photosensitizers have been applied in PDT for both malignant and non-cancerous disorders as a non-invasive therapy approach (28). And the most widely used photosensitizer is PpIX, which is induced from ALA exogenously administered.

On the one side, enabling to better protect the structure and function of underlying and surrounding tissues, ALA-PDT possesses a superficial effect that is ideally suited for the treatment of early-stage cancers. On the other side, its application is restricted to surficial tumors with insufficient effect in hypoxic areas, and requires interstitial optic fibers for nodules. As for the topical application, blue light (400 nm) can be employed because tissue penetration is just a few millimeters. Red light, which penetrates tissue more deeply than blue light and lasers connected to interstitial fibers are employed to achieve deeper penetration by delivery routes such as oral, intraperitoneal, intralesional, or instillation (29).

#### 3.2.1 ALA-PDT induced cell death

The capability of preventing the abnormal cell proliferation by inducing cell death including cell apoptosis and autophagy accounts for the high therapeutic effect of ALA-PDT (Figure 2). The process largely depends on the prior accumulation of PpIX in mitochondria, the power plant and pressure sensor of the cell. After being excited, PpIX is able to induce reactive oxygen species (ROS)—superoxide radicals, hydrogen peroxide, and hydroxyl radicals, which will subsequently impair the mitochondrial structure directly and cause the release of Ca^2+^ (30). As a result, the caspase-9-related apoptotic pathway in mitochondrial is activated by the mitochondrial membrane electric potential loss and further causes apoptotic cell death (31–33). Lipophilic PpIX, despite being synthesized in mitochondria, is able to be allocated to other intracellular regions, and then, promote cell apoptosis through photodamage effect, which so far has been found in the endoplasmic reticulum (ER) bringing about the release of Ca^2+^ and inducing cell apoptosis via ER stress response (34). Additionally, PDT-induced cell apoptosis may also involve caspase-irrelevant pathway as shown in the Hexyl-ALA mediated PDT (35).

Furthermore, closely related to ALA and irradiation dose, cell lines, and sites of PpIX, ALA-PDT can also cause cell necrosis. The cell apoptosis happens promptly in some cell strains in first few hours, while other cell strains are more likely to die from necrosis later on (36). The sites of PpIX also decide the manner of cell death. For example, when PpIX is located in mitochondria or the ER, ALA-PDT often promotes apoptotic pathways, but if PpIX is positioned in the plasma membrane, ALA-PDT frequently results in cell necrosis (37). Besides, PDT dose has influence on the cell death mode, as well. Cells tend to undergo apoptosis when exposed to low-dose PDT, and are prone to experience cell necrosis with high-dose PDT (38, 39).

Recently, ALA-PDT is reported to be involved with autophagy, the deterioration of organelles and cellular proteins, which actually does not cause tumor cells death but conversely assist tumor cells to confront chemotherapy and radiationtherapy (40). It has been verified by the fact that ALA-PDT mediated cell death can be reinforced when the cell autophagy is suppressed, which means cell autophagy somehow defends tumor cells from PDT (41–43). And the tuberous sclerosis complex 2 protein which can further induce the pro-survival autophagy is able to be inhibited by receptor-interacting protein 3 (RIP3) (44).

#### 3.2.2 ALA-PDT induced immune reactions

The cytotoxicity induced by ALA-PDT can activate inflammatory reactions and tumor-specific adaptive immune reactions (45), which has effect on the tumor-associated and tumor-infiltrating immune cells, therefore, induce multiple immune cells to infiltrate into the tumor microenvironment (Figure 2) (46). Despite the unconfirmed anti-cancer vaccine effect induced by PDT, is has been reported that ALA-PDT has the ability to mediate the production and release of specific damage-associated molecular patterns (DAMPs) through necrotic and apoptotic tumor cells which will further be identified by antigen-presenting cells (APCs) (13). As a result, APCs will submit tumor antigens to T cells activating tumor-specific immune reactions. By producing a proper microenvironment for anti-tumor immunity, PDT has become a particular therapy within other approved modalities. Though the exact mechanism of PDT-induced immune reactions remains undetected, it has been accepted that ALA-PDT is able to mediate the generation of anti-tumor immune reactions (47).

Additionally, ALA-PDT can also induce immunosuppression which inversely will lower the therapeutic effect of ALA-PDT on treating cancers involving the rise of regulatory T cells, the reduction of antigen-presenting Langerhans cells and the unleash of immunosuppressive cytokines (48–50). Therefore, to synergistically enhance the antitumor effect of ALA-PDT, the inhibitor of immune checkpoint protein, like death-ligand 1 (PD-L1) expressed by some squamous cancer cells, is needed to be used (49).

Studies have shown that PDT can significantly increase inflammatory cells such as regulatory T cells, plasmacytoid dendritic cells (PDCs), MHCII positive dermal DCs (51) and macrophages (52) after treatment in chronic wounds, thus promoting wound recovery. In addition, PDT is able to mediate the recruitment and differentiation of fibroblasts in chronic wounds (53). On the one hand, the remarkable increasement of transforming growth factor-β (TGF-β) expression after PDT treatment would probably induce myofibroblast differentiation in wound healing. On the other hand, it has been confirmed that PDT can facilitate mast cells (MCs) infiltration and the expression of a large number of fibroblast growth factor (FGF)—and consequently induces fibroblast recruitment and differentiation (54). Meanwhile, MCs is believed to serve chronic wound healing directly through transmitting signals to dermal DC, as well (55–58).

Current research has demonstrated the presence of neuroimmunomulation during wound healing (59–61). Sensory neurons possess the capability to enhance their membrane excitability via recognizing cytokines released by immune cells, thus activating mitogen-activated protein kinases (MAPK) and other crucial signaling pathways. Alternatively, they can perceive exogenous signals from the environment as well as endogenous danger signals emanating from tissue damage via distress signal receptors (62). Notably, studies have indicated that stimulation of the dorsal root elicits heightened inflammatory responses (62), encompassing chemotaxis and activation of inflammatory cells, degranulation of MCs, dendritic cell activation, and the differentiation of helper T cells (51, 63, 64).

The interaction between nerves and other cells involved in wound healing, such as MCs, plays a crucial role in the healing process (62, 65, 66). Studies have confirmed that the degranulation index of MCs increases after PDT for chronic wounds, promoting the release of nerve growth factor (NGF) and vasoactive intestinal peptide (VIP), which can activate neurons and nerve fibers in the dermis, thus promoting wound repair (67). Additionally, there is a significant increase in neuronal populations containing neurotransmitters related to wound healing, such as NGF, VIP, substance P (SP), and neuropeptide Y (NPY) (59, 60), which enhances the release of these neurotransmitters (68). The activation of nerve fibers may also be associated with increased secretion of extracellular matrix by fibroblasts (54), elevated levels of TGF-β (51), and cellular infiltration responses (54).

### 3.3 Photodynamic inactivation

In view of extensive antibiotic resistance and the increase of new infections, photodynamic inactivation (PDI) aiming at microbes—fungi, bacteria, and viruses has received more attention (Figure 2). The mechanism of PDI is parallel to that of general photodynamic applications: the exogenous irradiation, the generation of photosensitizer-induced ROS, and the damage caused by ROS. The targets of PDI contain: virus proteins, nucleic acids (DNA or RNA), and viral lipids, if it exists. The viruses having lipids are likely to be more susceptible in PDI because of the additional target (69). Though the influence of PDI for viral lipids has not been investigated yet (70), the researches on anticancer PDT has revealed the damage caused by ROS on viral lipids. Up to now, PDI has already been applied on two fields: the blood products purification and the HPV infection treatment.

## 4 Clinical applications

### 4.1 Cervical intraepithelial neoplasia

Cervical cancer is currently one of the most prevalent oncological diseases (71). According to the Global Cancer Observatory International Agency for Research on Cancer, 603,863 new cases of cervical cancer were reported in 2020. The absolute number of fatalities caused by this illness accounted for 7.7% of all cancer-related deaths in women (72). At the squamocolumnar junction of the cervix uteri, a pre-malignant state known as cervical intraepithelial neoplasia (CIN) can be seen, i.e., dysplastic uterine cervix changes that, if ignored and uncontrolled, is likely to develop into cervical cancer. Virtually all occurrences of cervical cancer and its predecessors have the human papillomavirus (HPV), mostly high-risk strains 16 and 18, locally infected as the essential cause (73). Over the past few decades, CIN has become more common, particularly among younger women. The diagnosis of CIN was used in clinical practice to identify individuals who had an elevated risk of getting cervical cancer (nearly 20 times higher in the case of HSIL) and use different treatment techniques to stop its progression.

#### 4.1.1 PDD of CIN

The identification of CIN lesions is now based on mass screening with cervical cytology and HPV testing, which is determined by national screening strategies. Colposcopy with acetic acid is performed after identifying a subject at risk to examine the position, size, and probable grade of the lesion; meanwhile we are able to obtain guided biopsies from it. Colposcopy has a high sensitivity but a significantly lower specificity. As a result, new approaches with a higher detection rate would be needed in order to define lesions more correctly.

In a study that randomly selected 68 women for the diagnosis of CIN, Hillemanns et al. contrasted colposcopy with 5-ALA-based photodiagnosis (74). The 0.5% or 1% aqueous solution of 5-ALA was applied to the cervix which were examined using a specialized endoscope coupled with a color filter and a spectrometer for semi-quantitative fluorescence image processing and spectrum analysis. It turned out that the fluorescence was inadequate for clinical application when used 0.5% solution of 5-ALA. And the ideal application period for 1% 5-ALA solution was determined to be 60–90 min. Photodiagnosis had similar sensitivity and specificity to colposcopy in terms of identifying CIN, with 94 and 51% vs. 95 and 50%, respectively. However, significantly, photodiagnosis showed a specificity of 75% for the same sensitivity.

For 73 patients (43 suffered from CIN and 30 as normal control without CIN), Szafiska-Dolata et al. contrasted colposcopy with 5-ALA-based photodiagnosis (75). For 4 h, a 3% 5-ALA gel was topically administered to the cervix. Through a light source (405 nm), fluorescence was stimulated. The outcomes of the direct biopsies, colposcopies, and photodiagnosis were compared to the conclusive histological diagnosis from endocervical curettage and direct biopsies. According to those findings, PDD increased the sensitivity (91%) and specificity (93%) of CIN identification compared to colposcopy (79%) and cytological diagnosis (43%), respectively. Photodiagnosis makes it possible to properly pinpoint the location of a CIN lesion and assess its length, boundaries, and multifocal nature.

Another similar study conducted by Vansevičiūtė et al. showed that photodiagnosis had the edges on identifying high-risk changes in CIN (76). They included 48 women who had been recommended for colposcopy due to high-grade alterations in cervical cytology; every cervix examined was divided into quadrants—174 quadrants total. For PDD, 3% 5-ALA cream was applied topically followed by 135 min incubation. The ratio of PpIX fluorescence intensity at 634 nm and autofluorescence intensity at 510 nm were assessed at each spectrum. According to the data, the PDD sensitivity for each patient was 91.2%, higher than that of colposcopy (88.2%). The authors indicated that PDD can be utilized to identify high-risk cervical intraepithelial neoplasia since accuracy was 85.4% and efficacy was more than 80%.

The 68 individuals evaluated by Nowakowski et al. had CIN alterations of varying degrees (77). Before PDD, 5-ALA cream (15%) was applied to the cervix for 2 h, after which the light with a wavelength of 400–420 nm was used to stimulate the fluorescence. And the results from cytology, colposcopy, PDD, and histology were associated in 41 individuals with low grade alterations and malignancy. In 27 patients with high grade alterations, the percentage of the histological results in line with colposcopic evaluation is 55.6% while the positive fluorescence impact of PDD consistent with was 63%. The authors further suggested that the effectiveness, specificity, and sensitivity of PDD are comparable to those of colposcopy in the diagnosis of serious neoplasia.

A double ratio (DR) fluorescence imaging approach was used in the research carried out by Bogaards et al. for noninvasive stage of CIN (78). To distinguish dysplastic tissue from the normal one, fluorescence spectroscopy was performed with two distinct excitation wavelengths. Localizing the regions where the CIN was detected colposcopically was easier with the aid of the intensity ratios determined through the light with two excitation wavelengths. It was observed that the value of DR found at the biopsy location corresponded statistically with the disease stage evaluated by histopathology.

Cervical biopsies taken after 1.5, 3, or 6 h of topical administration of benzoporphyrin derivative monoacid ring (BPD-MA) or 5-ALA acid were tested for CIN by Keefe et al. (79). With fluorescence microscopy and hematoxylin and eosin staining, the samples were examined for signs of selective drug accumulation. After being exposed to 5-ALA, cervical tissue had little stromal fluorescence and maximum fluorescence in dysplastic cells comparing to normal cells. Nevertheless, nonselective, diffusion-driven absorption was shown after the application of BPD-MA, which first emerged in the epithelium’s superficial cells and then the residual cells and stroma without selectivity. The fact that 5-ALA is specially absorbed by dysplastic cervical cells confirms that this substance is an effective photosensitizing prodrug for the PDT of CIN.

Before the cervix conization on account of CIN, Pahernik et al. (80) topically administered 10 mL of 3% 5-ALA. The incubation period lasted 1–6 h. The dysplastic epithelium of the ectocervix included many distinctive fluorescence (634 and 704 nm, PpIX-specific), while the stroma beneath the epithelium did not exhibit any fluorescence. Between CIN III lesions and healthy tissue, it was shown to be a considerable selectivity. After 180 min, the peak fluorescence for high-grade lesions was identified; reduced selectivity was seen for both longer and shorter application intervals.

#### 4.1.2 PDT of CIN

There are already several CIN therapy options available, all of which have been verified effectiveness. Techniques for excision that provide useful pathological specimens include cold knife conization and loop electrosurgical excision. However, postoperative bleeding is quite frequently related to them. Cryotherapy and laser ablation are destructive procedures, but they are typically constrained by inadequate postoperative colposcopy. The significant excision or destruction of cervical stroma is the main drawback shared by all treatments. Current treatments may result in cervical incompetence concerning premature amniotic membranes rupture, premature deliveries, and low birth weight babies or, negatively, scar stricture which will raise the risk of infertility and cesarean section (81, 82). The great mass of patients, who are still in their reproductive age, are looking for a noninvasive CIN therapy to prevent these dangers (83). Though HPV vaccines today are quite widespread, they cannot solve the issue. Since the existing HPV vaccinations are not therapeutic and only offer complete protection in those who have never had or are currently infected with HPV, CIN will continue to be a therapeutic problem for decades (84).

As an alternative to the foregoing issues, 5-ALA-mediated photodynamic treatment has been put into practice in a number of clinical studies which show that 5-ALA PDT is an effective and safe therapy for CIN. Ma et al. (85) evaluated the efficacy of 5-ALA-PDT for CIN II by conducting a clinical research enrolling 210 patients diagnosed as CIN II. Four hours after application of the 20% 5-ALA gel to the cervicovaginal and cervical canal, patients received PDT with light dose of 100 J/cm^2^ at 635 nm. As for the results, PDT group showed higher pathological regression rate (80/87, 92.0%) than cryotherapy group (79/97, 81.4%), as well as the HPV clearance rate.

Many studies also verify that 5-ALA PDT not only has good curative effect on CIN but also can be used for relieve persistent HR-HPV infection which will probably deteriorate into cervical cancer but with no effective therapy. A prospective study conducted by Fu et al. (86) applying three times 5-ALA-PDT (10% 5-ALA gel, 635 nm, 100 J/cm^2^) every 2 weeks showed that at 9 months follow-up, the complete remission rates of the treatment group was 76.92% (30/39) and 32.40% (12/37) for the control group. Furthermore, the conversion rates of abnormal ThinPrep cytology test (TCT) findings were, respectively, 90.90% (10/11) and 25.00% (2/8). Another clinical study using 6% 5-ALA gel and red light (635 nm, 200 J/cm^2^) to eliminate premalignant lesions of cervical—HPV infection and/or CIN I (87). The results were positive and satisfactory. After three treatments, 80% of patients with HPV only wiped the infection, meanwhile, 83% of patients diagnosed with HPV-associated CIN I had the infection eradicated. Similar results can be seen in the clinical research of Wang et al. (88) which evaluated the therapeutic efficacy of ALA-PDT for low-grade squamous intraepithelial neoplasia (LSIL) with HR-HPV infection. Irradiation (635 nm, 100 J/cm^2^) was performed to cervical surface and canal after incubation with 20% ALA gel for 4 h. At 6-month after PDT (three treatments with 7–14 days apart), 90.1% of patients achieved pathological regression, and 81.8% of patients eliminated HPV infection, at the same time, 78.0% of patients got a significant shrinking of cervical ectropion and a decrease in vaginal discharge.

As for high-grade squamous intraepithelial neoplasia (HSIL), Tang et al. (89) conducted a clinical research involving 99 patients who suffered from HSIL with HR-HPV infection. Similarly, ALA-PDT showed high effectiveness and safety. After receiving PDT (20% ALA gel, 635 nm, 80–100 J/cm^2^), HPV clearance rate and lesions complete remission (CR) rates were 81.3 and 92.5%, respectively, at 1-year follow-up. They further evaluated the efficacy of ALA-PDT in different groups presenting PAX1^hm^ (high methylation levels of PAX1 genes) or PAX1^lm^ (low methylation levels of PAX1 genes) which has been considered to have a strong correlation with the occurrence of cervical lesions (90–92). According to the results, the HPV clearance rate and CR rates of PAX1^lm^ group (71.2, 95.5%) were obviously higher than that of PAX1^hm^ group (36.8, 73.7%). Additionally, the average treatment times of PAX1^lm^ group was less than that of PAX1^hm^ group, which may be due in part to the high level of PAX1 methylation genes decreases the function in regulating immune system and reduces the therapeutic efficiency of ALA-PDT.

Wang et al. (93) compared the efficacy of ALA-PDT among different grades of CIN by a retrospective study of 183 patients who were confirmed with CIN histologically and received ALA-PDT (20% ALA gel, 635 nm, 80–100 J/cm^2^) which were conducted 4–6 times at an interval of 7–10 days. At 1-year follow-up, the HPV clearance and CR rate were 84.5 and 90.2%, which has no statistically significant differences between CIN I, CIN II and CIN III. Their results suggest that the high efficacy of ALA-PDT is unaffected by the grades of lesions.

Besides, ALA-PDT is not only a safe and feasible therapy, but also has advantages over other conventional treatments. Chen et al. (94) respectively evaluated the therapeutic effect and the complications of CO_2_ laser therapy (control group) and ALA-PDT (experimental group), which has been regarded to be a minimally invasive treatment. In the retrospective analysis, they selected patients confirmed HPV infected LSIL. Patients in the experimental group received ALA-PDT 3–7 days after menstruation with 23.6% ALA gel and 635 nm red light irradiation (144 J/cm^2^) once every 7–14 days for 3 times. At 6-month follow-up, the HPV clearance rate and LSIL reversal rate in PDT group were 79.0 and 80.6% which were much higher than those of CO_2_ laser group (62.3 and 64.2%). Moreover, the incidence of complications in experimental group was significantly less than that in control group. Their results indicate that ALA-PDT is a promising treatment with significant efficacy—better than CO_2_ laser therapy, and no scars left.

As the first choice for patients diagnosed with HSIL, Loop electrosurgical excision procedure (LEEP) will probably cause preterm birth defectively (95). Su et al. (96) conclude that ALA-PDT is not merely a non-invasive, effective and safe treatment, but also able to retain structure and function integrity of organs. In the retrospective analysis, they selected 48 patients diagnosed with CIN and vaginal intraepithelial neoplasia (VAIN). After receiving ALA-PDT (20% ALA gel, 635 nm, 80–100 J/cm^2^), The CR rate was 88.64% at 6-month follow-up. During follow- up, 4 patients achieved successful pregnancies, without any vaginal bleeding or discharge.

Apart from that, according to Cai et al. (97), ALA-PDT is capable of setting off immune response. ALA-PDT was performed on patients suffering CIN II (20% ALA solution; 635 nm, 100 J/cm^2^), and two patients were randomly chosen for observation of pathological sections before and after treatment. At 3-month follow-up, the total response rate was up to 90.77%, and CD4+ cells significantly increased at 6-month follow-up.

It has been reported that despite LEEP/CKC, the universal accepted treatment for HSIL, persistent HPV infection and subsequent recurrence still has been threatening many patients (98). Whereas, another surgery will bring about adverse complications—neighboring tissue damage and cervical adhesion which carry risks for prospective pregnancy including abortion and premature birth (99, 100). Therefore, Wang et al. (101) targeted the efficacy of ALA-PDT in patients suffered from persistent infection of HR-HPV after cervical conization (LEEP and CKC). The therapy used 20% ALA solution and deployed irradiation at 632.8 nm (100 J/cm^2^). One year after PDT, the total HPV clearance reached up to 88.24%, while the negative conversion rate of HPV16/18 was 76.00 and 95.35% for other HR-HPV. According to the results, ALA-PDT is reputed to be an effective and non-invasive treatments for patients with relentless HPV infection after traditional therapy, in order to circumvent the impair raised by secondary surgery.

In view of the advantages that ALA-PDT has, it has been applied to make up the deficiencies of clinically existing therapies. Cai et al. (102) evaluated the efficacy of ALA-PDT following LEEP in six women. They treated patients with persistent CIN following LEEP with ALA-PDT for 4–7 sessions (20% ALA solution; 635 nm, 120 J/cm^2^). The results showed that HPV clearance was 83% and the negative conversion rate was 100% at 6-month follow-up. They recommend ALA-PDT as an adjunct treatment to LEEP for patients with high-grade CIN, which will prevent recurrence to a large extent. Zhang et al. (103) conducted a retrospective analysis involving 83 patients confirmed with HSIL and positive margins after conization. Among them, 33 patients received ALA-PDT (20% ALA gel; 635 nm, 100 J/cm^2^), 15 patients had repeat conization 1 month after the initial treatment. In the analysis, the HPV clearance rates at 2-year follow-up were 78.95, 84, and 92.3% in control, ALA-PDT, and repeat conization groups respectively; while the recurrence rates were 26.1, 3.3, and 6.7%, respectively. Their results indicate that ALA-PDT is expected to be an effective treatment for women suffering high-grade CIN with a positive margin after cervical conization which is able to greatly reduce the recurrence rate.

### 4.2 Vulvar intraepithelial neoplasia

Vulvar Intraepithelial Neoplasia (VIN) contains two categories: vulvar high-grade squamous intraepithelial lesions (vH-SIL) and differentiated vulvar intraepithelial lesion (dVIN) (104). The prevalence of VIN in young women is increasing, and it frequently returns despite radical surgery, which can result in severe deformity. And the best way to treat these lesions is still up for debate.

Vulvar high-grade squamous intraepithelial lesions (vH-SIL), associated with HPV infection, usually afflict young women and are less likely to generate invasive malignancy (104, 105), which account for 86.7% of all VINs with a yearly incidence rate of 5 per 100,000 (106). In order to avoid recurrence, conservative therapy is necessary as long as possible (107, 108). It has been widely accepted that though a biopsy of HSIL, wide local excision will be carried out if occult cancer is questionable. Unfortunately, early detection and accurate positioning are still challenging. Specifically, the diagnosis relies on the visual inspection in the gynecological examination including the confirmation depending on small local incision biopsies (109), which cannot completely represent the size and stage of the tumor. Because of the less likely to progress to invasive cancer, therapy contains topical treatments, ablation, and a watch-and-wait approach.

Due to the development of differentiated vulvar intraepithelial lesion (dVIN) in pre-existing lichen sclerosus, older women are more susceptible (60–80 vs. 30–50 for vH-SIL). Its mechanisms so far encompass ischemic, p53 mutations, chronic inflammation, and oxidative stress (109, 110). Moreover, dVIN has a greater potential for malignant change than vH-SIL, which has been found mostly deteriorates to serious invasive vulvar squamous cell carcinoma (SCC) (111). Therefore, the primary for dVIN is surgical excision (cold knife excision or LEEP).

#### 4.2.1 PDD of VIN

So far, the interpretation of relief or pigmentation abnormalities is still reliant on the operator. Colposcopy is of value to figure out the extent of lesions which are not precisely delineated in women. Whereas, this examination can be useless because local inflammation and pruritus may disguise the HSIL lesions. Therefore, the risk to underestimate invasive cancer must be considered before treatment (112).

Photodiagnosis, a non-invasive technique, has been devised to make up the clinical examination by detecting the fluorescence generated from the photosensitizer preferentially accumulating in diseased tissues. Even tiny lesions can be detected by PDD, including the vulvar cancer in its early stages when it might form on the basis of irregular epithelium. However, colposcopy is hard to interpret lesions at that stage, especially when multifocal abnormalities develop.

Maździarz et al. (113) evaluated the efficacy of PDD using two concentrations of 5-ALA cream (3 and 15%) in the detection of vulvar lesions. Vulvar tissue of two groups was exposed to the irradiated light (405 nm) after being coated with 5-ALA cream for 4–6 h. According to their results, the sensitivity of both 3%-ALA group and 15%-ALA group were 100%, and the specificity of two groups were 92 and 91.4%, respectively. And the correlation with the histological examination of 3%-ALA group and 15%-ALA group were 93.9 and 93.4%. Researchers found that 3% 5-ALA cream is sufficient for photodynamic detection, but unfortunately, PDD is unable to discern cancer and dysplasia, which means the histopathological verification for suspected sample is necessary. Certainly, the superiority of PDD over other examination makes it possible to diagnose the disease precisely at a very early stage, and obtain a thorough knowledge of lesion topography which will finally facilitate confirming the extensiveness of surgery before the treatment.

Akoel et al. (114) assessed the precision of PDD in terms of identifying, positioning and distinguishing precancerous lesions and invasive cancer of the vulva. All patients received 5-ALA topical application for 3–6 h, followed by an irradiation with a wavelength of 380–440 nm. According to the results, PDD had accuracy and high efficiency in vulvar diseases diagnosis. For the identification of all VIN, the sensitivity, specificity, positive and negative predictive values were 92.9, 90.2, 91.2%, and 92.0, respectively.

Despite these positive findings, preclinical research is still not allowed to employ PDD as a routine structured vulvar HSIL screening. To bolster the ideal circumstances for enhancing the specificity and sensitivity of PDD-based vulvar HSIL, more research is necessary (115).

#### 4.2.2 PDT of VIN

VIN has been an increasingly common clinical problem in past years, especially among young women (116), probably on account of the increased occurrence of HPV- related high-grade squamous intraepithelial lesions (117). Despite reports of spontaneous regression, it is generally agreed that VIN should be aggressively treated owing to its possibility for invasion and related physical and psychological issues (118). Currently, surgical excision and CO_2_ laser ablation are the two most common therapies for VIN. However, genital mutilation and disfiguration will follow surgical excision in individuals with multifocal scar (119), to say nothing of high recurrence rate (119). Therefore, the point is to adopt a kind of conservative treatment which aims to preserve normal anatomy and function of vulvae, effectively eradicate lesions, and reduce the recurrence rate meanwhile.

Hillemanns et al. (107) compared the therapeutic effect of different treatments for VIN by conducting a retrospective evaluation involving 93 patients with histologic diagnosis of VIN then underwent various therapies. The PDT was carried out only once using 20% 5-ALA and an irradiation light at 635 nm (100 J/cm^2^). They found the complete response of PDT was only 52%, but it had the lowest rate of side effects. Through excisional therapy and vulvectomy were able to achieve higher complete response rate, they unfortunately could bring about sequelae, like vulvar mutilation which consequently caused psychological distress. However, it has been verified that PDT is capable of producing extremely good cosmetic outcomes while conserving healthy tissue and destroying sick tissue to a minimum. In another research, they drew the conclusion consisting with the results above (120). They also speculated that the low response rate of PDT was associated with increased pigmentation and hyperkeratosis of the lesion.

Booth et al. (121) assessed the effectiveness of PDT for VIN selecting patients who had no response to other treatments. They applied 5-ALA topically 4–6 h before irradiation (630 nm, 100 J/cm^2^). According to their results, most patients had milder symptoms after 2–3 weeks.

Additionally, the study directed by Fehr et al. (122) showed that PDT had similar effect to conventional treatments (laser evaporation and local excision) as well as additional edge in treating patients with VIN III—short healing time and better preservation of normal vulvar tissue. The 10% 5-ALA gel was applied to the entire vulva followed by exposure to irradiation light (635 nm, 120 J/cm^2^). At 2-month follow-up, there is no ulcers and scarring observed. And it showed no statistically significant difference among PDT group and two traditional therapy groups.

With respect to disparate effect of PDT, Choi et al. (123) conclude that the effectiveness of PDT for VIN is related to the PS administration. They had patients injected hematoporphyrin derivatives (2 mg/kg) intravenously, and after 48 h patients received irradiation light exposure (630 nm, 120 J/cm^2^). If the persistent disease occurred, the patient would get secondary PDT with topical 5-ALA. The results revealed that the CR rate was 80% 3 months after first PDT, and 71.4% at 1-year follow-up. They believe the skin folds and hair follicles may prevent local PS from sufficient penetration (124). Therefore, the defect is likely to be the topical agent administration rather than 5-ALA itself.

Later Zhao et al. (125) further figure out the solution of the issue. They combined 5-ALA PDT with superficial shaving as the treatment for patients who suffered from multifocal high-grade VIN and without any response to other therapies. Superficial shaving was carried out only once before the first PDT, and the procedure of 5-ALA PDT (20% 5-ALA cream; 633 nm, 120 J/cm^2^) was performed in three courses (1-week interval). At 1-year follow-up, 94% patients had clinical response, and 71% patients got remarkable cosmetic outcomes. In addition, the expression of p16 and Ki-67 significantly reduced after 5-ALA PDT, which indicates that PDT is able to induce changes in cell cycle and apoptotic proteins and the therapeutic effect derives from cytotoxic reaction in abnormal epithelial cells.

Previously, Fehr et al. (126) also mentioned that the PDT-induced selective lesion destruction was associated with not only direct cell apoptosis or necrosis (127) but also immune changes (128). In their research, the PDT procedure was performed with 10% 5-ALA gel and the irradiation dose was 80–125 J/cm^2^ (635 nm); in addition, their results showed that 5-ALA PDT appeared to be as effective as conventional therapies with shorter healing time and no scar formation.

### 4.3 Vaginal squamous intraepithelial lesion

Vaginal squamous intraepithelial lesion (VaIN) describes varied degrees of atypical hyperplasia only in the vaginal epithelial layer, typically prior to the development of vaginal epithelial carcinoma (129). The fact that VaIN is more prevalent in middle-aged and older women (130) may be associated with facts like the vaginal epithelium’s weakening, atrophy, and susceptibility to injury in consequence of changes in hormone levels with aging. Since its yearly incidence (0.2–2.0/100,000) is significantly less than that of CIN (270/100,000) (131), VaIN is frequently disregarded and the possibility of missed diagnosis is considerable (132). Fortunately, because the premalignant period of most patients is long and changeable, it is likely to cure patients completely once diagnosed and treated early (133). If not, approximately 10% of high-grade VaIN may deteriorate into vaginal invasive carcinoma (134). It has been reported that most patients with VaIN once have undergone cervical-associated surgery, and the majority of lesions are inspected during the screening or follow-up for CIN (135). And HPV persistent infection has been found to be the main factor inducing VaIN, especially HPV-16 (136). Nowadays, there have been several therapy modalities for VaIN including physical surgery, carbon dioxide laser, 5-fluorouracil, and radiation treatment. But the recurrence rate remains high, and the standard therapy for VaIN II-III is still absent (137–140).

#### 4.3.1 PDT of VaIN

Medical treatments now available including drugs, physiotherapy, and surgery are not effective for vaginal HSIL which may still be prone to recurrence. And they all lack long-term observation. The surgery may cause certain impact on the quality of patients’ life, while laser or cryotherapy do not have limited treatment depth and are likely to injure the surrounding organs such as bladder, urethra, and rectum. As a result, researchers tend to look for a novel therapy for VaIN.

Han et al. (141) evaluated the effectiveness and safety of 5-ALA PDT for high-grade VaIN involving 56 patients diagnosed with VaIN II/III. The PDT procedure was performed at 7–14 days intervals with 20% 5-ALA gel and the irradiation dose was 120–144 J/cm^2^ (635 nm). The results showed that at 6-month follow-up, the pathological regression rate achieved 87.5% and the HPV clearance rate was up to 41.9%. The adverse event observed during the treatment was increased vaginal discharge, which is not severe. The research affirmed the good curative effect and high security of PDT as a treatment for high-grade VaIN.

Cai et al. (142) also verified the conclusion above by directing a trial with 6 cases suffering HPV-induced VaIN. All patients received irradiation exposure (635 nm, 120 J/cm^2^) 3 h after the application of 20% 5-ALA solution. The PDT treatment was carried out in 4–8 sessions. And 4 of the 6 patients got HPV eliminated at 3–4 months after treatment. What’s more, most patients had recurrent trouble during the follow-up period. The authors concluded that combination with LEEP and 5-ALA PDT could obtain a satisfactory therapeutic effect on VaIN.

### 4.4 Condyloma acuminatum

Condyloma acuminatum (CA) or genital warts, a benign epithelial neoplasm induced by infection of HPV (mainly type 6 and 11), has been the most prevalent sexually transmitted disease (STI) worldwide (143). According to the World Health Organization, 101 million individuals worldwide suffer from CA each year, and the incidence rate ranges from 0.5 to 1% with an upward tendency year over year (144). Squamous cell carcinoma or VaIN can develop from partial CA, particularly if it has not been treated for a long period of time with frequent recurrence. Due to its connections to penile and cervical cancer, CA has also been identified as the national priority for prevention (145). The traditional therapies for CA include imiquimod, electrocautery, laser, and cryotherapy, in order to eliminate the visible lesions, but they all have a rather high recurrence rate (30–65%) which may be attributed to the fact that HPV can typically be found up to 1 cm away from the warts’ clinically obvious boundary (146). Additionally, people with impaired cellular immunity, such as Human Immunodeficiency Virus (HIV) infection or having transplantation, tend to response poorly to conventional treatments—more lesions and recurrence (147).

The high recurrence of CA is primarily on account of unsuccessful removal of HPV in subclinical and latent lesions. Therefore, the point is to identify all the lesions with HPV infection. According to some researches, 5-ALA induced PDD is able to distinguish the subclinical and latent lesions with specifical fluorescence.

Wang et al. investigated the effect of 5-ALA induced photo detection on CA diagnosis. They recruited 30 patients with genital warts and applied 20% 5-ALA cream to the lesions. After 2 h incubation, lesions were irradiated with LED light (410 nm), and the fluorescent PpIX emitted red fluorescence which clearly identified the lesion margins. The results showed that all the subclinical lesions had red fluorescence among which 70% spots got well-defined margin. Scientists believed that the nonspecific margin results from adjacent mucus, inflammation, and erosive lesion.

Inada et al. (148) also verified the feasibility of 5-ALA induced PDD on CA. They chose 40 women diagnosed with CA in different grades of lesions and topically applied 20% 5-ALA cream. After 6 h, the patients received emission light at 405 nm. It could be easily observed that the red fluorescence concentrated selectively on the clinical and latent lesions rather than healthy tissues.

#### 4.4.1 PDT of CA

For the time being, various treatments containing electrodesiccation and vaporization could merely eliminate the visible warts which means the persistent infection would still be the long-lasting risk of latent and subclinical lesions that induce the recurrence (149, 150).

The research of Hu et al. (151) verified that latent infection is more likely to be eliminated by three sessions of ALA-PDT. They applied 20% ALA gel on lesions and with a radius of 1 cm of the adjacent normal skin. After 3 h, the sites were exposed under the irradiated light with the wavelength of 635 nm (100 J/cm^2^). The results suggested that the ALA-PDT was capable of cleaning the HPV infection after 6 sessions (88.23%). Additionally, it was important to note that several patients with multiple sextual partners or the history of recurrence need close surveillance and monitoring. Especially, HPV genotypes and viral loads during PDT could be crucial indicators to guide the treatment (152). They also proved the conclusion above in another research (146). After PDT treatment, 93.33% of patients with latent or subclinical lesions got negative results on HPV tests, among which 80% had just one session of PDT. And all patients retained undetected until 6-month follow-up.

Romero et al. (153) also consider that the effectiveness of ALA-PDT for CA is more obvious in treating patients without immunodeficiency and lesions with more mucosa such as labia minora and labia majora.

Several studies compared the efficacy of ALA-PDT and traditional therapies. Buzzá et al. (154) conducted a randomized clinical trial to compare the effectiveness of PDT with trichloroacetic acid (TAA), an agent which is capable of destructing condylomas. The PDT was performed with 20% methyl aminolevulinate (MAL)—the methylated form of 5-ALA and irradiation light at 630 nm (100 J/cm^2^). The CR rate for TAA was 60 and 63.3% for PDT, however, they observed higher recurrence rate for TAA (33%) than PDT (0%). Besides, TAA and many other traditional therapies (imiquimod and surgical removal treatments) brought about side effects such as pain, ulceration, and crust formation (155), while PDT was able to treat latent infection and protect normal tissue construction without scarring at the same time.

The therapeutic effect of ALA-PDT in pregnancy was also evaluated. Yang et al. (156) contrasted the effectiveness of ALA-PDT with cryotherapy in treating pregnant women suffering from *CA.* Patients in PDT group received 3 rounds treatment (20% ALA solution; 633 ± 10 nm, 90–100 J/cm^2^), meanwhile, the cryotherapy group had 2 cycles of freezing and thawing for three times. It was reported that the wart clearance rate for PDT group was 93.8% but 72.7% for cryotherapy group. As for recurrence rate at 3-month follow-up, the PDT group was 6.3% while the cryotherapy group was up to 36.4%. Finally, all newborns developed well with no abnormality in physical examinations.

As for the mechanism of ALA-PDT therapeutic effect, Xie et al. (157) confirmed that the ALA-PDT induced immunity response in treating *CA.* According to their results, CD4+ and the expression level of IFN-γ mRNA increased at 4 h after PDT and later decreased at 24 h after PDT. Plasmacytoid dendritic cells (pDCs) showed a rising trend, and CD3+ was observed to infiltrate to the superficial dermis. Additionally, patients with significant increase of IFN-α and IFN-ß mRNA expression levels after treatment needed fewer rounds of PDT. The results above verify that ALA-PDT is able to induce T lymphocyte mediated, DC and pDCs related immunity. And the clinical effect of PDT in treating CA is possibly related to the rising levels of IFN-α and IFN-ß after treatment.

## 5 Conclusion and discussion

As we mentioned before, 5-ALA is not a photosensitizer, but the precursor of PpIX which has the fluorescent characteristic and subsequently causes photodynamic reactions. After selective absorption and accumulation in the abnormal cells, the transformed PpIX is going to emit red fluorescence (600–700 nm) with exposure to the light at a wavelength of 405 nm. As a result, here comes the application—photodynamic diagnosis (PDD). Compared with traditional inspection methods, ALA-PDD has various edges which make it a promising mean of detection for numerous diseases.

Since the diagnosis of early and precancerous lesions is of great significance on the treatment, a novel supplementary diagnostic approach is demanded. As a non-invasive detection method, ALA-PDD is able to point out malignant and precancerous lesions with high accuracy, especially the multifocal lesions, which will help to clearly define the boundary of lesions and facilitate the presurgical decisions. Additionally, ALA-PDD also contributes to the performance of needle aspiration biopsy. All these above shows that ALA-PDD has advantages on enhancing the accuracy of early-stage lesions verification. Some conventional tests that are now useful rely mostly on the experience and skill of the doctor who interprets the results, which may increase chances of mistakes and misdiagnosis. Restrictions also come from the cover with pubic hair, the layer of keratin, pigmentation deposition, the presence of squamous hyperplasia, etc., which bring difficulties for diagnosis by traditional detection methods. And ALA-PDD, is not expected to completely substitute the preceding tests, but can become a proper complement to them.

Though ALA-PDD shows great edges on diseases diagnosis, the effectiveness varies considerably, which may partially relate to the concentration of administrated ALA and its application time. At first, abnormal hyperplasia tissues will absorb the 5-ALA selectively, and then, the PpIX is transformed and accumulated in the cell. Later on, the PpIX becomes saturated. Theoretically, the blood concentration of PpIX is fairly low (158), which is the same to the concentration in the benign tissues. Despite the extremely slow uptake of 5-ALA in the normal cell, it still has a little absorption, which will definitely increase as time goes on or the 5-ALA concentration is high. In other words, as the duration of application time elongates or the 5-ALA concentration rises, the risk of PpIX accumulation in benign tissues increases. Therefore, the significant difference between lesions and normal tissues is hardly been seen. According to Xu et al. (159) the best setting for ALA-PDD of CIN is 20% 5-ALA and the administration time is 6 h. However, the protocol of other diseases still remains undetected.

In terms of Choi et al. (123), the ideal therapy for the lower genital tract precancerous lesions is supposed to meet the following conditions: (i) long-term CR with symptoms disappearance, (ii) the minimal invasive route, (iii) preservation of normal tissue and physiological structure, (iv) repeatable treatment without accumulated injury, and (v) suppression of multifocal lesions, and PDT just meets the above standards showing promising development prospect in treating numerous HPV-related or precancerous lesions. Compared to conventional therapies, with the equal or even better therapeutic effect, ALA-PDT, a greatly selective minimally invasive therapy, combines medicine and machines and is able to successfully prevent the deterioration of precancerous lesions preserving the normal structure and function to an overwhelming degree by non-invasive modality with laser and photosensitizer (160). Because of the mild side effects without accumulated injury, ALA-PDT could become a repeatable treatment which also shows better effectiveness with repeating sessions (89). Above all, ALA-PDT is suitable for multifocal lesions as well and can effectively avoid the relapse. Li et al. (161), also pointed out that PDT significantly improved complete remission, reduced recurrence, exhibited rapid and sustained HPV clearance compared to other therapies, which confirmed that PDT offered a promising, non-invasive treatment approach for HPV-associated female lower reproductive tract diseases through meta-analysis.

In view of multiple therapeutic effect of ALA-PDT in numerous researches, we have found some factors that affect the curative efficiency of ALA-PDT. Firstly, the action depth is a key point. Since 5-ALA may penetrate the whole epithelial layer, while 635 nm laser has a penetration depth of 3–5 mm, only lesions that are within the action depth of ALA-PDT are able to be treated effectively. For example, CIN and VaIN are restricted to the cervical and vaginal epithelium respectively, and their thickness is not beyond the ALA-PDT action depth, which to some extent ensure the curative effect (162). This is also embodied in the different therapeutic effect concerning various local physiological conditions. For instance, Zhao et al. (125) firstly combined superficial shaving with ALA-PDT in treating multifocal high-grade VIN and the results showed better effect than other researches that do not have the pubic hair shaved or even treat hyperkeratinic lesions (120). The ineffective penetration may account for such unsatisfactory effect. On the contrary, mucosa is probably to increase the penetration of M-ALA, which induces better therapeutic effect (153). Therefore, it is necessary to figure out highly efficient 5-ALA application conditions suitable for various positions and diseases.

Secondly, the protocol of 5-ALA application and treatment times may also affect the therapeutic effect of ALA-PDT. Since the accumulation of PpIX in abnormal cells is closely related to the concentration and administration time of 5-ALA, searching for an optimal choice for 5-ALA application is undoubtedly crucial, which consequently will affect the treatment outcomes. It has been verified that too low concentration or short administration time of the drugs probably reduce the accumulation of PpIX in lesions and finally causes ineffective PDT (87). Up till now, most clinical studies apply 20% 5-ALA with incubation time of 4–6 h. However, there is no specific criterion for ALA-PDT in treating gynecological disease, which needs to be decided in the future. In some reports, though the 5-ALA concentration is up to 20–30%, the effect is still dissatisfactory. We believe the point should be the improper dosage form. Despite the high drug concentration, 5-ALA aqueous solution presents insufficient effectiveness compared with 5-ALA gel or dispersant. Sometimes, the lesions with local anatomy variation, like atypical vessels, or patients with persistent HPV infection need more times of ALA-PDT to increase lesion removal probability and therapeutic effect. And it has been verified that receiving ALA-PDT only once has disappointing effect with poor remission rate (163).

Additionally, the recurrence of HPV infection and the immunity of patients can also have influence on the efficacy of ALA-PDT. It has been reported that the HPV reinfection raises the risk of relapse. Although the HPV test is negative at the end of ALA-PDT treatment, patients are required to improve their immunity, and have the follow-up and monitor of viral load.

Nowadays, almost all the clinical use of ALA-PDT involved red light with wavelength around 635 nm, but PpIX gets higher absorption peak at blue light (around 417 nm). Despite the deeper penetration of red light, ALA-PDT with blue illumination achieves basically equivalent therapeutic effects to that with red illumination in treating basal cell carcinomas (BCC) lesions at 6-month follow-up (164). And sometimes, the shallower penetration may also result in less adverse effects. This suggests that it is worthy to attempt to apply blue light induced ALA-PDT on the treatment of female lower genital tract diseases, which on the other hand promote the integration of ALA-PDD and ALA-PDT.

Since ALA is able to mediate PDD and PDT, it is promising to combine the disease diagnosis and treatment, which is called theranostics (165). Fluorescent theranostic drugs are compounds that can preferentially localize in the target tissue, showing it by characteristic fluorescence emission and motivating therapeutic activity in response to light activation. The selective tumor distribution of PpIX after 5-ALA application, along with PDT induced cytotoxicity, makes it a fluorescent theranostic drug for diagnostics and treatment of malignant lesions. The ALA-induced theranostics has been applied in a clinical experiment with patients suffering from glioblastoma who receive PpIX fluorescence-guided resection and ALA-PDT at once upon the same dose of 5-ALA (166). Efficiently combining ALA-PDD and ALA-PDT, ALA-induced theranostics may also become the prevailing trend providing hopeful progression-free survival in treating gynecologic lesions.

In conclusion, ALA-PDT has become an appropriate choice for young women with physiological and psychological needs, and red laser mediated ALA-PDT is being perfected further and popularized or put in practice step by step in many countries. In 2022, Chinese expert consensus on the clinical applications of aminolevulinic acid-based photodynamic therapy in female lower genital tract diseases has been published (167). However, this preliminary attempt is too ambiguous to carry out clinically. For example, it lacks the specific irradiation dose for each targeted lesion, and the testing protocols for the post-treatment follow-up period is absent, either. They are also the major issues which have been currently facing in practice. According to the researches mentioned in this article, the effective irradiation dose (630 ± 5 nm) is 80–100 J/cm^2^ for CIN, 100–120 J/cm^2^ for VIN, 120–144 J/cm^2^ for VaIN, and 90–100 J/cm^2^ for CA, which is much more definite than the general irradiation dose for all the female lower genital tract diseases (60–150 J/cm^2^) in the expert consensus. In addition, the testing protocol should also be added, which is often been carried out including TCT, colposcope and HPV-DNA test at least in the previous research. Hu et al. (168) have verified the therapeutic effectiveness of ALA-PDT in treating CIN2, and all 22 patients included are only aged 18. It would be more convincing if patients of a wider age range were enrolled in the study.

## Author contributions

YC: Data curation, Investigation, Methodology, Writing – original draft. PG: Data curation, Investigation, Project administration, Supervision, Validation, Visualization, Writing – review & editing. LC: Conceptualization, Funding acquisition, Project administration, Software, Supervision, Writing – review & editing. DH: Conceptualization, Funding acquisition, Methodology, Project administration, Software, Supervision, Visualization, Writing – review & editing.
